# Evolving Accelerated Amidation by SpyTag/SpyCatcher to Analyze Membrane Dynamics

**DOI:** 10.1002/anie.201707623

**Published:** 2017-12-05

**Authors:** Anthony H. Keeble, Anusuya Banerjee, Matteo P. Ferla, Samuel C. Reddington, Irsyad N. A. Khairil Anuar, Mark Howarth

**Affiliations:** ^1^ Department of Biochemistry University of Oxford South Parks Road Oxford OX1 3QU UK

**Keywords:** membrane proteins, protein engineering, protein–protein interactions, SpyTag/SpyCatcher, synthetic biology

## Abstract

SpyTag is a peptide that forms a spontaneous amide bond with its protein partner SpyCatcher. This protein superglue is a broadly useful tool for molecular assembly, locking together biological building blocks efficiently and irreversibly in diverse architectures. We initially developed SpyTag and SpyCatcher by rational design, through splitting a domain from a Gram‐positive bacterial adhesin. In this work, we established a phage‐display platform to select for specific amidation, leading to an order of magnitude acceleration for interaction of the SpyTag002 variant with the SpyCatcher002 variant. We show that the 002 pair bonds rapidly under a wide range of conditions and at either protein terminus. SpyCatcher002 was fused to an intimin derived from enterohemorrhagic *Escherichia coli*. SpyTag002 reaction enabled specific and covalent decoration of intimin for live cell fluorescent imaging of the dynamics of the bacterial outer membrane as cells divide.

Thousands of non‐covalent protein–protein interactions mediate cellular function. However, engineering covalent interactions between protein partners brings a range of new opportunities for basic research and synthetic biology.^1^ We have developed the use of spontaneous amide bond formation by peptide tags as a simple, specific, and genetically‐encoded route to lock protein units together.^2^ This technology, particularly the SpyTag/SpyCatcher pair, has been used in diverse applications including biomaterials, next‐generation sequencing, enzyme stabilization, and vaccine development.1a, 3 A key limitation has been relatively slow reaction at cellular expression levels. We established an evolutionary approach to achieve a second‐generation, faster‐reacting version of this protein superglue. We then applied the enhanced properties for efficient and specific cell‐surface functionalization, to investigate the outer‐membrane dynamics of intimin, a protein relevant to human colonization by pathogenic bacteria.

Since the SpyTag/SpyCatcher system is an unconventional approach to peptide interaction, it is likely that there are features of the interaction that cannot be predicted by rational design. Selection from phage libraries has been established for decades and the difficult thing is usually to detect weak interactions,^4^ rather than the challenge of screening for irreversible interactions.1b, 5 We established a panning procedure to select for covalent bond formation between SpyTag variants and the SpyCatcher bait (Figure 1 a, see Supporting Information for detailed methods). Key features we found to enable successful panning were: 1) capturing site‐specifically biotinylated SpyCatcher bait in solution, rather than attaching SpyCatcher to beads, 2) TEV protease cleavage to elute phage specifically from beads, and 3) washes harsh enough to dissociate non‐covalent interactions, but retaining phage infectivity (1× glycine pH 2 and 4× Tween‐20). For model selection, we incubated M13 phage displaying SpyTag on pIII with either reactive bait (SpyCatcher) or the negative control SpyCatcher EQ.2a Using this panning procedure we obtained 4 orders of magnitude enrichment for the specific covalently reacting partner (Figure 1 b).

**Figure 1:**
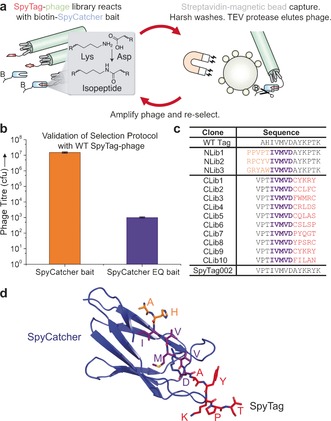
Selection of peptide for accelerated amidation. a) Cartoon of panning to select faster SpyTag variants displayed on pIII of M13 phage. Biotin is represented by B and streptavidin by small circles. b) Model selection for reactive peptide. SpyTag‐phage recovered after selecting with wild‐type SpyCatcher bait, compared with the non‐reactive SpyCatcher EQ bait, quantified as colony forming units (cfu) (mean±SD, *n*=3). c) Selected amino acid sequences of SpyTag clones from the final rounds of selection of the N‐terminal library (NLib1‐3) and the subsequent C‐terminal library (CLib1‐10). Residue colored orange if varied in the N‐terminal library, purple if not varied, and red if varied in the C‐terminal library. d) Structure of SpyCatcher in blue complexed with SpyTag (based on PDB ID: 4MLI), colored as in (c) .

Since mutating central residues in SpyTag abolished SpyCatcher reactivity,2a we made two different libraries, randomizing at the N‐terminal or C‐terminal ends of SpyTag (Figure 1 c,d). After panning, NLib1 (PPVPTIVMVDAYKPTK) gave the fastest reaction, with the first two residues able to be removed without affecting the rate (Figure S1a in the Supporting Information). The sequence VPT‐ was used thereafter at the N terminus, while the C terminus was randomized based on this lead. After rounds of phage library screening, the enriched hits CLib1‐10 are shown (Figure 1 c), with their position on the parent structure indicated (Figure 1 d).^6^ Of these variants, CLib1 (identified in two separate clones, also as CLib9) was fastest for reaction with SpyCatcher and preserved the YK pair at residues 9–10 of WT SpyTag. However, the cysteine residue in CLib1 was undesirable because of potential dimerization, so this residue was reverted to alanine (Figure 1 c). Addition of the terminal lysine of SpyTag (not present in the phage library) further increased the reaction rate. With this combination of phage selection and rational design, we arrived at the optimized SpyTag002 (Figure 1 c).

We established phage‐display selection of SpyCatcher similarly to SpyTag (Figure 2 a). Additional features important for successful SpyCatcher selection were: 1) a DsbA signal sequence for co‐translational translocation of SpyCatcher‐pIII^7^ and 2) growing in the XL‐1 Blue *E. coli* strain at 18 °C. For model selection, the bait was biotinylated Avitag‐SpyTag‐MBP (Figure 2 a), which showed an approximately 1000‐fold enhanced capture of WT SpyTag bait compared to non‐reactive SpyTag DA bait2a (Figure 2 b). The sequence of selected clones is indicated in Figure 2 c. Mutations were widely distributed over the structure, with many mutated residues distant from the SpyTag binding site (Figure 2 d). Hits were expressed as soluble proteins in *E. coli* and evaluated for speed of reaction with SpyTag‐MBP. The fastest reacting sequence was L1C6 (Figure 2 c and S1 b).

**Figure 2:**
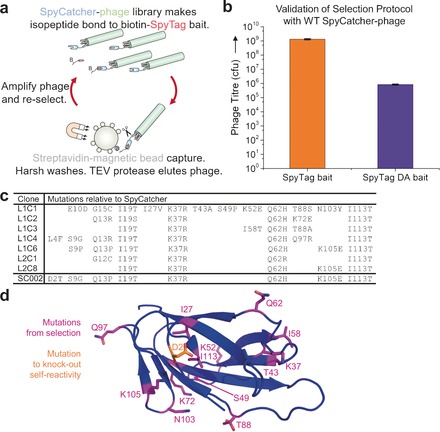
Selection of protein for accelerated amidation. a) Cartoon of panning for faster SpyCatcher variants. B represents biotin and the small circles are streptavidin. b) Model selection for SpyCatcher panning. SpyCatcher‐phage was selected with WT SpyTag‐MBP or the non‐reactive SpyTag DA‐MBP and quantified as cfu (mean±SD, *n*=3). c) Amino acid sequences of selected clones from the final round of SpyCatcher library selections. The final selected SpyCatcher002 is at the bottom (SC002). d) SpyCatcher mutations mapped on to the crystal structure. Selection‐derived mutations from WT SpyCatcher are in purple. Orange marks the mutation to inhibit self‐reactivity (structure of CnaB2 domain in PDB ID: 2X5P truncated at the end of the SpyCatcher002 sequence).

During this process, a new band was identified by sodium dodecyl sulfate polyacrylamide gel electrophoresis (SDS‐PAGE) after recombinant expression of L1C6 SpyCatcher (Figure S2 a). Since this band completely shifted upon mixing with SpyTag002‐MBP and had a mobility approximately twice that of SpyCatcher, we suspected that the band represented a covalent SpyCatcher–SpyCatcher dimer. We hypothesized that enhancing SpyCatcher reactivity had promoted unintended self‐reactivity. The N‐terminal GAMVDT of SpyCatcher resembles VMVDA of SpyTag (Figure S2 b). Mutation of GAMVDT to GAMVTT in our final variant (SpyCatcher002, Figure 2 c) removed this side reaction (Figure S2 a). Differential scanning calorimetry showed that the mutagenesis had a minimal effect on the thermostability: the melting mid‐point was 48.5 °C for SpyCatcher and 49.9 °C for SpyCatcher002 (Figure S3 a).

Upon characterizing the SpyTag002/SpyCatcher002 reaction, we confirmed the role of the putative reactive residues: single mutation in SpyTag002 (DA) or SpyCatcher002 (EQ) abolished reaction (Figure 3 a). SpyTag002 and SpyCatcher002 reacted under a wide range of pH (Figure 3 b) and temperature (Figure 3 c) conditions, following second‐order kinetics (Figure S3 b). Reaction was relatively independent of buffer salts (Figure S3 c), tolerating common non‐ionic detergents (Figure S3 d) and over 3 m urea (Figure S3 e). SpyCatcher002 reacted to 99 % completion with a small excess of SpyTag002‐MBP. Conversely, SpyTag002‐MBP reacted to 97 % completion with an excess of SpyCatcher002 (Figure S4). Loss of water upon SpyTag002/SpyCatcher002 reaction was confirmed by mass spectrometry (Figure S5).

**Figure 3:**
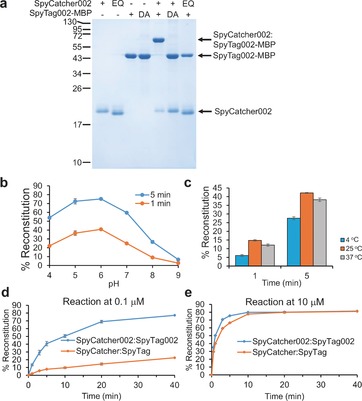
Characterization of spontaneous amidation between SpyCatcher002 and SpyTag002. a) Selective covalent bond formation. SpyCatcher002 and SpyTag002‐MBP were mixed at 10 μm for 1 h in succinate/phosphate/glycine buffer at pH 7.0 and analyzed after boiling by SDS‐PAGE with Coomassie staining. Unreactive control proteins, SpyCatcher002 EQ and SpyTag002 DA‐MBP are also shown. b) pH‐dependence of reaction of SpyCatcher002 with SpyTag002‐MBP for 1 or 5 min at 25 °C in succinate/phosphate/glycine buffer. c) Temperature‐dependence of reaction of SpyCatcher002 with SpyTag002‐MBP in phosphate‐buffered saline (PBS) pH 7.5. d) Time‐course for reaction of SpyCatcher002‐sfGFP with SpyTag002‐MBP (blue) or reaction of SpyCatcher‐sfGFP with SpyTag‐MBP (orange) at 0.1 μm in succinate/phosphate/glycine at pH 7.0. e) Reaction as in (d) but with 10 μm of each protein. Data show the mean±SD of triplicate experiments; some error bars are too small to be visible.

To analyze reactions at low concentrations (0.1 μm), we fused SpyCatcher to the N terminus of superfolder GFP. A major enhancement of reaction rate was seen with SpyTag002 and SpyCatcher002 compared to the parental versions (Figure 3 d). As expected, the difference was less marked as the concentration of both partners was increased to 10 μm, but the 002 versions were still faster (Figure 3 e). At 25 °C at pH 7.0, SpyTag002‐MBP reacted with SpyCatcher002 with a rate constant of 2.0±0.2×10^4^ m ^−1^ s^−1^ (12 times faster than SpyTag‐MBP reacting with SpyCatcher: 1.7±0.4×10^3^ m ^−1^ s^−1^). The new variants showed backwards compatibility, reacting efficiently with parental versions (SpyTag002 with SpyCatcher: 1.0±0.06×10^4^ m ^−1^ s^−1^; SpyTag with SpyCatcher002: 5.5±0.03×10^3^ m ^−1^ s^−1^; all given as the mean±SD of a triplicate experiment). SpyCatcher002 also behaved well as a C‐terminal fusion, as indicated by efficient reaction of MBPx‐SpyCatcher002 with SpyTag002‐MBP (Figure S6 a). Similarly, SpyTag002 reacted efficiently when fused either to the N terminus as SpyTag002‐MBP (Figure 3) or to the C terminus as AffiEGFR‐SpyTag002 (Figure S6 b).

We explored the use of the new reactive pair for analysis in living cells. Enterohemorrhagic *E. coli* O157:H7 is a common cause of food poisoning and can be lethal in children or the elderly. These bacteria express the virulence factor intimin in their outer membrane.^8^ It is a significant challenge to investigate the outer‐membrane proteins of Gram‐negative bacteria because fluorescent‐protein fusions are not functionally exported there.^9^ We used an intimin fusion to display SpyCatcher002 on the outer membrane of *E. coli* (Figure 4 a).^8^, ^10^ We showed specific labeling of SpyTag002‐sfGFP on bacteria expressing intimin‐SpyCatcher002 by live‐cell fluorescence microscopy (Figure 4 b). Consistent with the specificity of the SpyTag002/SpyCatcher002 interaction, the non‐reactive DA mutant of SpyTag002 did not label the cells (Figure 4 b). The specificity of the reaction of SpyTag002 or SpyCatcher002 on cells was further supported by western blotting (Figure S7). Labeling of intimin measured by flow cytometry was effective at lower concentration of fluorescent‐protein fusion and was faster when using SpyTag002/SpyCatcher002, compared to the original SpyTag/SpyCatcher fusions (Figure S8).

**Figure 4:**
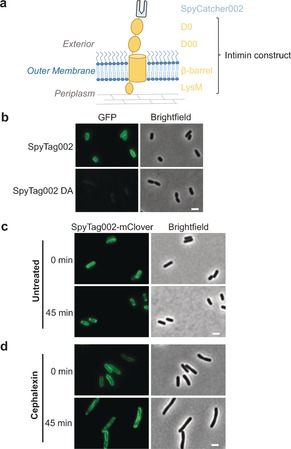
Application of covalently reacting partners to study bacterial outer‐membrane dynamics. a) Cartoon of the intimin‐SpyCatcher002 construct. The intimin construct contains a periplasmic domain mediating binding to peptidoglycan (LysM), a β‐barrel membrane‐spanning domain, and two immunoglobulin‐like domains (D00 and D0) before SpyCatcher002. b) *E. coli* expressing intimin‐SpyCatcher002 were labeled with SpyTag002‐sfGFP or the non‐reactive DA control and imaged by wide‐field fluorescence. GFP (green) and brightfield (grayscale) images are shown. c) *E. coli* expressing intimin‐SpyCatcher002 were labeled with SpyTag002‐mClover and imaged after 0 min (top row) or 45 min (bottom row) at 37 °C. mClover (green) and brightfield (grayscale) images are shown. d) Cells imaged as in (c) but after cephalexin treatment for 90 min. Scale bar: 2 μm.

We then set out to visualize the dynamics of the intimin fusion in response to cell division. Immediately post‐labeling, intimin‐SpyCatcher002 labeling was homogeneously distributed on the bacterial surface (Figure 4 c). After 45 min, the signal was distributed towards the bacterial poles (Figure 4 c, Movies S1, S2 in the Supporting Information), which is consistent with the trafficking properties shown for nutrient transporters in *E. coli*.^9^, ^11^ We reasoned that this polar movement results from incorporation of newly synthesized peptidoglycan, preparing bacteria for division. Therefore, we treated cells with cephalexin, a cephalosporin that blocks cell division by inhibiting peptidoglycan fusion at the division septum. Immediately after labeling, cells were elongated, consistent with inhibited division, and fluorescence was widely distributed on the outer membrane. After 45 min, localized patches of fluorescence were clearly visible and the bi‐polar localization was abrogated (Figure 4 d, Movies S3, S4), which is consistent with outer‐membrane protein movement being driven by helical and interspersed addition of peptidoglycan.^9^ The polar localization with and without cephalexin is quantified in Figure S9.

In summary, we were able to adapt phage display to select for faster spontaneous amidation, thereby enhancing both SpyTag and SpyCatcher reactivity. SpyTag002 has 4/13 residues that are different to those in SpyTag and an extra residue at the N‐terminus. SpyCatcher002 had 8/116 residues that are different to those in SpyCatcher: seven to increase reaction rate and one to remove a site of SpyTag similarity. SpyTag002 and SpyCatcher002 demonstrated rapid reaction under a wide range of buffers, temperatures, and pH values, and as N‐terminal or C‐terminal fusions. SpyTag002/SpyCatcher002 allowed specific covalent pulse‐labeling of surface proteins on living cells and represents the fastest currently available Tag/Catcher pair.2b, 12 In future work it will be important to test these new variants for challenging in vitro labeling, such as coupling antigens at high density on virus‐like particles for vaccination.^13^ Our removal of self‐reaction may be important for SpyCatcher002‐nanoparticles, so that rare intersubunit reaction does not promote aggregation. In addition, SpyTag has found application in vivo, for example, for imaging in *C. elegans* 14 or super‐resolution microscopy,^15^ so the rate acceleration here may bring further benefits. Our selection approach may also be valuable for evolving other binding technologies, as synthetic biology moves beyond conventional protein–protein interfaces.1b

## Conflict of interest

M.H., A.H.K., and S.C.R. are authors on a patent application covering sequences for enhanced isopeptide bond formation (UK Intellectual Property Office 1706430.4).

## Supporting information

As a service to our authors and readers, this journal provides supporting information supplied by the authors. Such materials are peer reviewed and may be re‐organized for online delivery, but are not copy‐edited or typeset. Technical support issues arising from supporting information (other than missing files) should be addressed to the authors.

Supplementary

Supplementary

Supplementary

Supplementary

Supplementary
