# Supplementary material for: Evolving Accelerated Amidation by SpyTag/SpyCatcher to Analyze Membrane Dynamics
Source: Angew Chem Int Ed Engl. 2017 Dec 5;56(52):16521–5. doi: 10.1002/anie.201707623 (PMC5814910; doi:10.1002/anie.201707623)
Supplement: Supplementary file 1 — Supplementary [file ANIE-56-16521-s001.pdf]

Angewandte Chemie
Eine Zeitschrift der Gesellschaft Deutscher Chemiker

Supporting Information

Evolving Accelerated Amidation by SpyTag/SpyCatcher to Analyze Membrane Dynamics

*Anthony H. Keeble+, Anusuya Banerjee+, Matteo P. Ferla, Samuel C. Reddington, Irsyad N. A. Khairil Anuar, and Mark Howarth**

anie_201707623_sm_miscellaneous_information.pdf
anie_201707623_sm_Movie_S1.avi
anie_201707623_sm_Movie_S2.avi
anie_201707623_sm_Movie_S3.avi
anie_201707623_sm_Movie_S4.avi

Author Contributions

A.K. Conceptualization: Equal; Data curation: Equal; Formal analysis: Equal; Investigation: Lead; Methodology: Lead; Resources: Equal; Supervision: Supporting; Writing – original draft: Equal; Writing – review & editing: Equal
A.B. Conceptualization: Equal; Data curation: Equal; Formal analysis: Equal; Investigation: Equal; Methodology: Equal
S.R. Conceptualization: Supporting; Investigation: Supporting; Methodology: Supporting; Resources: Supporting
M.F. Conceptualization: Supporting; Data curation: Supporting; Formal analysis: Supporting; Investigation: Supporting; Resources: Equal
M.H. Conceptualization: Equal; Funding acquisition: Lead; Project administration: Lead; Supervision: Lead; Writing – original draft: Equal; Writing – review & editing: Equal
I.K. Formal analysis: Supporting; Investigation: Supporting; Resources: Supporting.

Supporting Information

Table of Contents

General Methods
Cloning of constructs
Generation of a randomized N-terminal library of SpyTag
Generation of a randomized C-terminal library of SpyTag
Generation of libraries of SpyCatcher variants by error-prone PCR
Production of phage
Purification of phage by precipitation
Panning of library variants
Expression and purification of proteins
Isopeptide bond reconstitution experiments
Mass Spectrometry
Differential scanning calorimetry
Structure visualization
Bacterial labeling
Microscopy
Western blotting
Flow cytometry
Supporting references
Figures S1-S9
Legends for Movies S1-S4

General Methods

Cloning of constructs

Q5 High-Fidelity Polymerase (NEB) was used to perform PCR reactions and site-directed mutagenesis. Gibson Assembly Master Mix (NEB) was used following the manufacturer's instructions. All constructs were initially cloned into chemically competent *E. coli* NEB5α cells (NEB).

pET28a SpyTag-MBP (Addgene plasmid ID 35050), pET28a SpyTag DA-MBP, pDEST14 SpyCatcher (GenBank JQ478411, Addgene plasmid ID 35044), and pDEST14 SpyCatcher EQ (Addgene plasmid ID 35045) have been described previously.[S1] Both the pET28a and pDEST14 plasmids result in the SpyTag and SpyCatcher proteins being expressed with N-terminal His_6-tags. pDEST14 Avitag-SpyCatcher (GenBank accession no. KU500645, Addgene plasmid ID 72326) with WT and EQ versions, containing a peptide tag (Avitag) for site-specific biotinylation,[S2] was constructed from pDEST14 SpyCatcher or pDEST14 SpyCatcher EQ using SLIM PCR[S3] with primers 5'-GATTACGACATCCCAACGACCGAAAACCTG, 5'-GCCTGAACGATATTTTTGAAGCGCAG AAAATTGAATGGCATGAAGGCGATTACGACATCCCAACGACCGAAAACCTG, 5'-GTGATGGTGATGGTGATGGTAGTACGACATATG and 5'-TGCCATTCAATTTTCTGCGCTTCAAAAAT ATCGTTCAGGCCGCTGCCGTGATGGTGATGGTGATGGTAGTACGACATATG. pET28a Avitag-SpyTag-MBP and Avitag-SpyTag DA-MBP were constructed by inserting the same biotinylation tag (but without the TEV protease cleavage site) into pET28a SpyTag(WT/DA)-MBP using 5'-ATTACATATGGGTCTGAATGATATTTT CGAAGCGCAGAAAATTGAATGGCATGAAGGTAGCGGAGCCCACATCGTGATGGT G and 5'-GGGGAAGCTTTTACGAGCTCGAATTAGTCTG. The insert was digested with HindIII (NEB) and NdeI (NEB) and ligated into pET28a. pET28a Avitag-SpyTag002-MBP was constructed from pET28a Avitag-SpyTag-MBP via a two-stage Gibson assembly.

Individual SpyTag variants (including pET28a SpyTag002-MBP, GenBank MF974389 and Addgene plasmid ID 90001) were created using QuikChange with pET28a SpyTag-MBP as template. Mutants SpyCatcher002 EQ (Addgene plasmid ID 89998) and SpyTag002 DA-MBP (Addgene plasmid ID 90002) were constructed by QuikChange with the same mutations as previously.[S1] Selected SpyCatcher variants were cloned from the pFab5cHis phagemid vector to pDEST14 for the expression of soluble protein using PCR amplification of the SpyCatcher variant with forward (5'-CCGAAAACCTGTATTTTCAGGGCGCCATG) and reverse (5'-GCATCAACCATTTAGCTACCACTGGATCC) primers. The reverse primer retains the GSGGS peptide linker of pFab5cHis C-terminal to SpyCatcher, to allow subsequent overlap with the pDEST14 vector. Additional point mutations in selected SpyCatcher variants (including pDEST14-SpyCatcher002, GenBank MF974388 and Addgene plasmid ID 89997) were introduced by QuikChange.

pET21 MBPx-SpyCatcher (N-terminal His_6 tag–MBPmt–spacer–MBPmt–spacer–SpyCatcher) (GenBank accession no. KU361183, Addgene plasmid ID 72327) was previously described.[S4] pET21 MBPx-SpyCatcher002 was generated via three-part Gibson assembly. SpyCatcher002 was amplified from pDEST14-SpyCatcher002 using forward (5'-CGAGCTCGGGTTCGGGCGGTAGTGGTGCC ATGGTAACCACCTTATCAGGTTTATCAGGTG) and reverse (5'-GTGGTGGTGCTCGAGTG CGGCCGCAAGCTTCTATTAAGTATGAGCGTCACCTTTAGTTGC) primers. The template backbone was generated in two parts from pET21 MBPx-SpyCatcher using four primers (5'-GGTTTCGCCACCTCTGACTTGAGCGTCG; 5'-

CATGGCACCACTACCGCCCGAACCCGAGCTCG, 5’-AAGCTTGCGGCCGCACTCGAGCACCACCACCACCACCACTGAGATCCGGC; 5’-CGACGCTCAAGTCAGAGGTGGCGAAACC) and connected by Gibson assembly. pET28a AffiEGFR-SpyTag002 (with SpyTag002 at the C-terminus of an affibody to EGFR)[S4-5] was generated via two-part Gibson assembly using four primers (5’-GGCAGCATTGAATTTATTAAAGTGAACAAAGGCAGTGGTGAGTCG GGATCCGGAGCTAGC; 5’-GTTTATTATTTATAGCGTTTGTAGGCGTCCACCATAACAATAG TAGGAACACCGGAACCTTCCCCGGATCCCTCGAGGCC; 5’-GGACGCCTACAAACGCTATA AATAATAAACTCTAGCACCACTGAGATCCGGCTGCTAAC; 5’-ACTGCCTTTGTTCACTTTA ATAAATTCAATGCTGCCCAGTTTCCCCATATGGCTGCCGCG), with pET28a SnoopTag-AffiEGFR-SpyTag (GenBank accession no. KU296973) as the template.[S5] pET28a His_6-MBP was created by cloning the maltose binding protein gene from pMAL (NEB) in to the pET28a vector as previously described.[S1] pRK793 encoding MBP-TEV protease[S6] was modified by removing the TEV cleavage site in the spacer between MBP and the TEV protease.

pJ404 SpyCatcher-sfGFP encoding SpyCatcher fused to superfolder GFP (sfGFP)[S7] was a kind gift from Karl Brune (University of Oxford) and was produced in a three-part Gibson assembly. SpyCatcher (including the His_6-tag and TEV protease cleavage site) was amplified from pDEST14-SpyCatcher using forward (5’-GTTTAACTTTAATAAGGAGATATACCATGTCGTACTACCATCACCATCACC) and reverse (5’-CTTTACGGCCTGAACCACCAATATGAGCGTCACCTTTAGTTGC) primers. sfGFP preceded by a GGSG linker was amplified with forward (5’-GGTGGTTCAGGCCGTAAAGG) and reverse (5’-CCTTGGGGCTCGAGTTAT CATTTGTACAGTTCATCCATACCATGC) primers from pJ404-sfGFP (DNA2.0). The plasmid backbone was amplified using forward (5’-CATGGTATATCTCCTTATTAAAGTTAAACAAAATTATTTCTACAGGG) and reverse (5’-TGATAACTCGAGCCCCAAGG) primers. The three PCR products were then linked by Gibson assembly. pJ404 SpyCatcher002-sfGFP was created by amplifying SpyCatcher002 from pDEST14-SpyCatcher002 using forward (5’-CATGGTATATCTCCTTATTAAAGTTAAACAAAATTATTTCTACAGGG) and reverse (5’-TGATAACTCGAGCCCCAAGG) primers. The vector backbone was amplified in two parts using four primers (5’-GGTGGTTCAGGCCGTAAAGGCGAAGAGCTG; 5’-CGCGATTTGCTGGTGACCCAATGCGACCAGATGCTCCACGCCCAGTCGCGTACCG TCCTC; 5’-GCCCTGAAAATACAGGTTTTCGGTCGTTGGG; and 5’-GAGGACGGTACGCGACTGGGCGTGGAGCATCTGGTCGCATTGGGTCACCAGCAA ATCGCG) and the final construct was produced by Gibson assembly.

pQE SpyTag002-sfGFP was created from pQE SpyTag-sfGFP, a kind gift from Karl Brune (University of Oxford), using staggered QuikChange[S8] with primers 5’-TGTTATGGTGGACGCCTACAAACGCTATAAAGGATCAGAAAACCTGTATTTTCAG GGAGG and 5’-TTCTGATCCTTTATAGCGTTTGTAGGCGTCCACCATAACAATAGTAGGAACCATA TGTTTTACCTCCTAAAAGTTAAACAAAATTATTTC. pQE SpyTag002 DA-sfGFP was created from pQE SpyTag002-sfGFP by staggered QuikChange using 5’-GTTATGGTGGCCGCCTACAAACGCTATAAAGG and 5’-GTTTGTAGGCGGCCACCATAACAATAGTAGGAAC.

pET28 SpyTag002-mClover3 was constructed from pKanCMV mClover3-mRuby3, which was a gift from Michael Lin (Addgene plasmid ID 74252).[S9] The C-terminus of

mClover3 (GITHGMDELYK), which was missing in the fusion, was reconstructed by assembly PCR using the internal primers 5'-GAGCCCTTGTACAGCTCGTCCATGCCATGTGTAATCCCGGCGGCGGTCACGAACTCCAGC and 5'-ATGGTGATGGTGATGGTGGGAGCCGGAGCCGGAGCCCTTGTACAGCTCGTCCATGCCATG and external primers 5'-ATGGTGATGGTGATGGTGGGAGCCGGAGCCGGAGCCCTTGTACAGCTCGTCCATGCCGAG and 5'-AACCTGTATTTTCAGGGAGGTGGTTCAGGCGTGAGCAAGGGCGAGGAGCTGTTCACCGGG. pET28 SpyTag-mClover3 was constructed from pET28 SpyTag002-mClover3 by Gibson assembly.

pET28 intimin was made by synthesis of the gene encoding amino acids 1-659 from *E. coli* O157:H7 intimin[S10] and cloning into pET28. To make pET28 intimin-SpyCatcher002 (GenBank MF974390), SpyCatcher002 was amplified from pET28 SpyCatcher002 and inserted into pET28 intimin by Gibson assembly. This cloning generated a C-terminal myc tag for antibody recognition. pET28 intimin-SpyCatcher002 EQ was derived from pET28 intimin-SpyCatcher002 by staggered QuikChange and contained a point mutation of the key glutamate,[S1] blocking reaction with SpyTag002. pET28 intimin-SpyCatcher was made from pET28 intimin by Gibson assembly.

The phagemid plasmid was a variant of pFab5cHis encoding a PelB leader sequence, a cloning site and the C-terminal domain of M13 gIII.[S11] pFab5cHis SpyTag-gIII was created by inserting SpyTag between PelB and gIII: the plasmid was digested with XhoI and NotI and primers 5'-TCGAGGGCGGCGCCCACATCGTGATGGTGGACGCCTACAAGCCGACGAAGGGCGC and 5'-GGCCGCCTTCGTCGGCTTGTAGGCGTCCACCATCACGATGTGGGCGCCGCCC were annealed and ligated into pFab5cHis. To generate pFab5cHis SpyTag DA-gIII, pFab5cHis was digested with XhoI and NotI. Primers 5'-TCGAGGGCGGCGCCCACATCG TGATGGTGGCCGCCTACAAGCCGACGAAGGGCGC and 5'-GGCCGCCTTCGTCGGCTTGTAGGCGGCCACCATCACGATGTGGGCGCCGCCC were annealed and ligated into the vector.

pFab5cHis SpyCatcher-gIII was constructed in a two-step process. In the first step, SpyCatcher followed by the TEV cleavage site GSSGSENLYFQGSG was cloned in-frame with PelB and gIII in pFab5cHis. SpyCatcher was amplified from pDEST14 SpyCatcher using 5'-TAATCTCGAGATCAGGGCGCCATGGTTGATACCTTATC and 5'-ATATGCGGCCGCTCCACTCCCCTGGAAGTAGAGGTTTTC. The insert and vector were digested using XhoI and NotI and then ligated. In the second step, the PelB signal sequence was replaced with the DsbA leader sequence[S12] by SLIM PCR using 5'-GCGTTTAGCGCATCGGCGGGCAGCTACCCATACGATGTTCCAGATTACGCTGGTGCAGCTGCAGGTCG, 5'-CGCCGATGCGCTAAACGCTAAAACTAAACCAGCCAGCGCCAGCCAAATC TTTTTCATAGCTGTTTCCTGTGTGAAATTG, 5'-GGTGCAGCTGCAGGTCG, and 5'-TTTCATAGCTGTTTCCTGTGTGAAATTG.

All mutations and constructs were verified by sequencing. Multiple sequence alignments were generated using Clustal Omega.

Generation of a randomized N-terminal library of SpyTag

The library was assembled from one PCR-amplified fragment of pFab5cHis SpyTag-gIII and one restriction-digested vector by ligation. The insert was amplified by PCR using forward (5'-ACCTCGAGATNNKNNKNNKNNKNNKATCGTGATGGTGGACGCCTACAAGCC)

and reverse (5’-ATTCATATGGTTTACCAGCGCCAAAGACAAAAGGG) primers flanking the SpyTag section, adding *XhoI* and *NdeI* sites. DpnI was added to the insert PCR mixture following thermal cycling and incubated at 37 °C for 1 h, with heat-inactivation at 80 °C for 20 min. Vector DNA was digested with XhoI and NdeI at 37 °C for 1.5 h and heat-inactivated at 65 °C for 20 min. Total insert and vector reaction mixtures were mixed with 6× DNA loading dye and separated by agarose gel electrophoresis. DNA bands corresponding to the vector and insert were purified by gel extraction. Insert DNA was digested with XhoI and NdeI at 37 °C for 1 h and heat-inactivated at 65 °C for 20 min. Digested insert was cleaned and concentrated using a Thermo Scientific spin column and eluted in MilliQ water. Ligation was performed at the optimized vector:insert molar ratio of 1:7 (1:1 weight) with 627 ng DNA of each fragment in a total volume of 150 µL. DNA and water were heated to 65 °C for 5 min, cooled, T4 DNA ligase (NEB) and buffer were added, and the mix was incubated at 25 °C for 1 h. DNA was concentrated on a spin-filter and transformed into electrocompetent ER2738 amber stop-codon suppressor cells (Lucigen) by electroporation. Transformants were recovered by addition of 950 µL SOC medium at 37 °C for 1 h and plated on LB agar, containing ampicillin at 100 µg/mL and tetracycline at 25 µg/mL. Plates were incubated at 37 °C for 16 h. To harvest the library, 5 mL LB was added to the plate surface and cells were scraped with a plastic spreader and pipetted into a 50 mL Falcon tube. This procedure was repeated with another 5 mL LB. After collecting from all plates, the cells were pelleted at 2,500 g for 10 min at 4 °C and resuspended in 10 mL LB containing ampicillin (100 µg/mL), tetracycline (25 µg/mL) and 22% (v/v) glycerol. Aliquots were flash-frozen and stored at -80 °C.

Generation of a randomized C-terminal library of SpyTag

The library was assembled from two PCR-amplified fragments of pFab5cHis SpyTag-gIII. In the first PCR, the forward primer (5’-CGACCTCGAGATGTGCCTACTA TCGTGATGGTGGACNNKNNKNNKNNKNNKGCGGCCGCAGGCTCTAAAGATATC AGACC) converts the N-terminus of SpyTag to start with the residues VPT instead of AH, in addition to introducing the C-terminal mutations. The reverse primer started from the ampicillin resistance gene (5’-GATCGTTGTCAGAAGTAAGTTGGCC). In the second PCR reaction, the forward primer primed from the ampicillin resistance gene (5’-GGCCAACTTACTTCTGACAACGATC) and the reverse primer (5’-GTCCACCATCACGATAGTAGGCACATCTCGAGGTCGACCTGC) was from the start of the VPT-SpyTag, immediately prior to the region being mutated. The two PCR products were digested with DpnI as above, mixed with DNA loading dye, and separated by agarose gel electrophoresis. DNA bands were purified by gel extraction and joined by Gibson assembly. DNA was concentrated and transformed into electrocompetent ER2738 cells.

Generation of libraries of SpyCatcher variants by error-prone PCR

The libraries were assembled from two PCR-amplified fragments from pFab5cHis SpyCatcher-gIII by Gibson assembly. The vector was amplified using KOD polymerase (EMD Millipore) with oligonucleotide primers flanking SpyCatcher (forward primer: 5’-GGATCCAGTGGTAGCGAAAACC; reverse primer: 5’-AACCATGGCGCCCTGATCTCG). The insert was amplified with Taq polymerase under error-prone conditions (0.4 mM $MnCl_2$; 0.24 mM dGTP, 0.2 mM dATP/dCTP/dTTP) with forward primer 5’-CCTCGAGATCAGGGCGCCATGG and reverse primer 5’-GAAGTAGAGGTTTTCGCTACCACTGGATC for 18-23 cycles (varied to alter the mutational load on SpyCatcher). DpnI was added following thermal cycling, incubated at 37 °C for 1 h, and heat-inactivated at 80 °C for 20 min. Total reaction mixtures were mixed with 6× DNA loading dye and separated by agarose gel electrophoresis. DNA bands for the vector

and insert were purified by gel extraction (Thermo Scientific) and linked by Gibson assembly. DNA was concentrated and transformed into electrocompetent XL1 Blue amber stop-codon suppressor cells (Agilent Technologies).

Production of phage

Libraries of SpyCatcher in XL1 Blue and SpyTag in ER2738 cells were converted to phage-displayed protein libraries by infection with R408 helper phage (Agilent). For the first round of panning, a larger phage grow-up was required, using 250 mL 2xTY. Ampicillin (100 μg/mL), tetracycline (25 μg/mL) and 0.2% (v/v) glycerol were also included for production of SpyCatcher phage. This medium was inoculated with 100 μL of -80 °C library culture stock for the cells produced from the initial libraries as described above. For subsequent panning rounds, 600 μL of -80 °C library culture stock was used to inoculate 100 mL of the growth medium. For purification of monoclonal phage variants, overnight starter cultures (cultured in the growth medium) were used to inoculate (at a 1:100 dilution) 15 mL of growth medium. Cultures were grown at 37 °C at 200 rpm until OD_{600} 0.5 (~3-4 h), infected with 10^{12} R408 helper phage, and incubated at 80 rpm at 37 °C for 30 min. Expression of SpyCatcher/SpyTag-pIII was induced with IPTG (0.42 mM for SpyTag phage production and 0.1 mM for SpyCatcher phage) and incubated for 18-20 h at 200 rpm at either 25 °C (SpyTag) or 18 °C (SpyCatcher).

Purification of phage by precipitation

Infected bacterial cultures were centrifuged at 15,000 g for 10 min at 4 °C to remove the bacterial cells. One volume of precipitation buffer [sterile, 20% (w/v) PEG8000, 2.5 M NaCl] was added to 4 volumes of supernatant. The supernatants were mixed and incubated at 4 °C for 3-4 h. Phage were pelleted by centrifugation at 15,000 g for 30 min at 4 °C and the supernatant was removed. Phage pellets were resuspended in PBS (137 mM NaCl, 2.7 mM KCl, 10 mM Na_2HPO_4, 1.8 mM KH_2PO_4) pH 7.5 (2 mL per 100 mL culture) and centrifuged at 15,000 g for 10 min at 4 °C to clear any residual cells, before the supernatant was transferred to a new tube. The mixture was precipitated again as previously, but this time resuspended in 0.25 mL PBS per 100 mL culture. Samples were centrifuged at 15,000 g for 10 min at 4 °C and phage were precipitated a third time and resuspended in a final volume of 0.25 mL PBS per 100 mL culture. Samples were stored short-term (1-2 weeks) at 4 °C, or long-term at -80 °C. Typically, a 100 mL culture gave 250 μL of 10^{12} phage/mL.

Panning of library variants

Biotinylated Avitag-SpyCatcher was used as bait to react with SpyTag phage libraries. Biotinylated Avitag-SpyTag-MBP was used as bait to react with SpyCatcher phage libraries. The non-reactive bait variants (biotinylated Avitag-SpyCatcher EQ and biotinylated Avitag-SpyTag-DA-MBP) were included in parallel selections to assess the efficiency of the panning. Reactions were carried out in PBS pH 7.5 with 3% (w/v) bovine serum albumin (BSA) and supplemented with 25 μM His_6-MBP (for SpyCatcher phage selections to counter-select for SpyCatcher variants that bind to MBP) at 25 °C. In the first panning round, 1×10^{12} phage were mixed with 0.5 μM bait and reacted for either 5 h (SpyTag-phage) or 18 h (SpyCatcher-phage). Two subsequent rounds of panning were carried out for SpyTag-phage (0.2 μM biotinylated Avitag-SpyCatcher and 30 min reaction in round 2; 0.2 μM biotinylated Avitag-SpyCatcher and 10 min reaction in round 3). For the round 3 reaction, we included 10 mM dithiothreitol to remove any disulfide-linked adducts. For SpyCatcher-phage, three subsequent selection rounds were carried out (0.2 μM bait and 30 min reaction in round 2; 0.2 μM bait and 10 min reaction in round 3; 0.05 μM bait and 10 min reaction in round 4). The time of reaction was controlled by adding excess (50-100 μM) bait without an Avitag.

Phage were purified from unreacted biotinylated bait by precipitation. The pellet containing the phage-biotinylated bait adduct was resuspended in PBS pH 7.5 0.1% (v/v) Tween-20. 200 µL phage were mixed with 25 µl Biotin-Binder Dynabeads (Thermo Fisher Scientific) in a 96-well low bind Nunc plate that had been pre-blocked for 2 h at 25 °C with 3% (w/v) BSA in PBS pH 7.5 + 0.1% (v/v) Tween-20. The beads were captured using a 96-well microtiter plate magnetic separation rack (NEB) and washed 4 times with 200 µL/well PBS pH 7.5 + 0.1% (v/v) Tween-20.[S13] For each well in the microtiter plate, beads were resuspended in 200 µL PBS pH 7.5 0.1% (v/v) Tween-20 containing the phage-biotinylated bait adduct and incubated at 800 rpm for 1 h at 25 °C. To remove weakly bound phage, beads were washed at 25 °C, once with 150 µL glycine-HCl pH 2.2, then four times with 150 µL TBS (50 mM tris-hydroxymethyl aminomethane + 150 mM NaCl, pH 7.5) with 0.5% (v/v) Tween-20. Phage were eluted from beads by TEV protease digestion at 34 °C for 2 h at 1,000 rpm in 50 mM Tris•HCl pH 8.0 with 0.5 mM EDTA using 50 µL 0.72 mg/mL MBP-TEV protease. Eluted phage were rescued by infection of 1 mL of mid-log (OD_{600} = 0.5) cultures of ER2738 (for SpyTag-phage) or XL-1 Blue (for SpyCatcher-phage). Functional display of SpyCatcher on phage was assisted by the DsbA signal sequence.[S12] Cells were grown in LB supplemented with 25 µg/mL tetracycline at 37 °C at 80 rpm for 30 min. The cells were then diluted into 100 mL 2xTY [supplemented with 1% (v/v) glucose, 100 µg/mL ampicillin and 25 µg/mL tetracycline] and grown for 12-16 h with shaking at 200 rpm. After addition of glycerol to 20% (v/v), aliquots were flash-frozen and stored at -80 °C. The number of phage eluted was quantified by plating serial dilutions.

Expression and purification of proteins

SpyCatcher variants were expressed in *E. coli* C41 DE3,[S14] a gift from Anthony Watts (University of Oxford). All other proteins were expressed in *E. coli* BL21 DE3 RIPL (Stratagene). Single colonies were picked into 10 mL LB containing either 100 µg/mL ampicillin (pDEST14) or 50 µg/mL kanamycin (pET28a) and grown overnight at 37 °C with shaking at 200 rpm. 1 L LB supplemented with 0.8% (w/v) glucose and appropriate antibiotic in ultra-yield baffled flasks (Thomson Instrument Company) was inoculated with 1/100 dilution of the saturated overnight culture and grown at 37 °C with shaking at 200 rpm. After reaching OD_{600} 0.5-0.6, the cultures were inoculated with 0.42 mM IPTG and incubated at 30 °C with shaking at 200 rpm for 4-5 h. Cells were harvested and lysed by sonication in TBS containing mixed protease inhibitors (Complete mini EDTA-free protease inhibitor cocktail, Roche) and 1 mM PMSF and purified by Ni-NTA (Qiagen). Proteins were dialyzed into PBS with three buffer changes using 3.5 kDa molecular weight cut-off Spectra/Por dialysis tubing (Spectrum Labs). MBP-TEV protease was expressed and purified in a similar manner, except the protein was dialyzed three times in 50 mM Tris•HCl pH 8.0 + 0.5 mM EDTA. Avitag biotinylation with GST-BirA was performed as described.[S15] Protein concentrations were determined from OD_{280} using the extinction coefficients from ExPASy ProtParam.

Isopeptide bond reconstitution experiments

Isopeptide bond formation was monitored as previously described.[S1] Buffers used were: HEPES [50 mM 4-(2-hydroxyethyl)-1-piperazine pH 7.5], HBS (50 mM HEPES + 150 mM NaCl pH 7.5), TBS, PBS, PBS + 1 mM EDTA (ethylenediamine tetraacetic acid) pH 7.5. Reactions were quenched by adding 6× SDS-PAGE loading dye [0.23 M Tris HCl pH 6.8, 24% (v/v) glycerol, 120 µM bromophenol blue, 0.23 M SDS], followed by heating at 95 °C for 6 min in a Bio-Rad C1000 thermal cycler. Reactions were analyzed using SDS-PAGE on 16% polyacrylamide gels using the XCell SureLock system (Thermo Fisher Scientific) at 200 V with staining using InstantBlue (Expedeon) Coomassie. Band intensities were quantified using a Gel Doc XR imager and Image Lab 5.0 software (Bio-Rad). Percentage isopeptide

reconstitution was calculated by dividing the intensity of the band for the covalent complex by the intensity of all the bands in the lane and multiplying by 100. The second-order rate constant for covalent complex formation was determined by monitoring the reduction in intensity of the band for the SpyCatcher partner relative to a control not incubated with the SpyTag partner, to give the concentration of unreacted SpyCatcher partner. Time-points were analyzed during the linear portion of the reaction curve. 1/[SpyCatcher variant] was plotted against time and analyzed by linear regression using Excel (Microsoft) and Origin 2015 (OriginLab Corporation).

For measuring concentration-dependent rates (Figure 3d and 3e), SpyCatcher-sfGFP or SpyCatcher002-sfGFP was used. The reaction was quenched at 50 °C after addition of SDS-loading buffer, to retain the fluorescence of sfGFP. Reactions were run on 16% SDS-PAGE and the unreacted covalent product bands were quantified using a Fluorescent Image Analyzer FLA-3000 (FujiFilm) and ImageGauge version 4.21 software.

Temperature-dependence was measured in PBS pH 7.5 (since its pH has only a small variation with temperature) with 0.5 μM of each protein. For pH-dependence, each protein was mixed at 0.5 μM and 25 °C in succinate–phosphate–glycine buffer (12.5 mM succinic acid, 43.75 mM NaH_2PO_4, 43.75 mM glycine; pH was adjusted using HCl or NaOH), enabling suitable buffering over a broad pH range. Buffer-dependence was measured in PBS (± 1 mM EDTA), HBS, HEPES, or TBS at pH 7.5 with 0.5 μM of each protein at 25 °C. Detergent-dependence was measured with 0.5 μM of each protein at 25 °C in PBS pH 7.5 supplemented with 1% (v/v) Tween-20 or 1% (v/v) Triton X-100.

Assays to test SpyCatcher002 and SpyTag002 reaction to completion were carried out in succinate–phosphate–glycine buffer at pH 7.0 for 1 h at 25 °C, with one partner at 10 µM and the other partner at 10 or 20 µM.

Assays to test SpyCatcher002 reaction with SpyTag002-MBP in increasing concentrations of urea were carried out in PBS including the required concentration of urea (from 0-8 M), which was subsequently adjusted to pH 7.5 using HCl. All reactions were carried out using freshly prepared urea-containing buffer solutions at 2 μM of each protein in triplicate at 25 °C. The extent of reaction was analyzed after 30 min and 120 min.

Mass spectrometry

95 µM SpyCatcher002 was reacted with 220 µM peptide containing SpyTag002 (KGVPTIVMVDAYKRYK, solid-phase synthesized by Insight Biotechnology at >95% purity) for 3 h at 25 °C in PBS pH 7.5. The reaction was dialyzed against 10 mM ammonium acetate pH 7.5 using 3.5 kDa cut-off Spectra/Por dialysis tubing (Spectrum labs) three times each for 3 h at 4 °C. Mass spectrometry was performed using a Waters LCT Premier XE (Waters Corporation) equipped with electrospray interface, after the sample had been passed through a Merck Chromolith C18 2 × 5 mm guard column. The software used to analyze the data and convert the m/z spectrum to a molecular mass profile was MassLynx 4.1 (with OpenLynx open access) (Waters Corporation). The predicted molecular mass of the covalent complex was calculated using ExPASy ProtParam, taking into account the cleavage of N-terminal fMet and subtracting 18 Da for isopeptide bond formation.

Differential scanning calorimetry

Experiments were performed with 30 µM SpyCatcher or SpyCatcher002 in PBS pH 7.5 on a VP Capillary DSC (Malvern). Thermal transitions were monitored from 20 to 100 °C at a scan rate of 1 °C/min at a pressure of 3 atm. Data were analyzed using MicroCal DSC Origin Pro 7.0 software (Malvern). After the buffer (PBS pH 7.5) blank was subtracted from the experimental sample and the values were corrected for concentration and volume, the baseline

was subtracted. Subsequently the observed transition was fitted to a non-two-state model to obtain the melting temperature (T_m) and Full Width Half Maximum.

Structure visualization
Protein structures were rendered in PyMOL (DeLano Scientific), based on Protein Data Bank files 2X5P[S16] and 4MLI.[S17]

Bacterial labeling
E. coli BL21-T7Express (NEB) transformed with intimin-SpyCatcher002 was grown overnight in LB with 50 µg/mL kanamycin at 37 °C at 200 rpm. In the morning, 50 µL culture was diluted into 3 mL M9 minimal media [filtered and autoclaved solution comprising 4.7 mM Na_2HPO_4, 2.2 mM KH_2PO_4, 1.85 mM NaCl and 1.8 mM NH_4Cl; then separately sterilized solutions with indicated final concentrations were added just before use – 0.1 mM $CaCl_2$, 5 mM $MgCl_2$ and 0.4% (w/v) glucose] with 50 µg/mL kanamycin and grown to OD_{600} 0.2-0.4, before inducing with 0.42 mM IPTG at 37 °C at 200 rpm. After 3 h, 2 µM SpyTag002-mClover3 was added to the culture for 15 min at 25 °C at 200 rpm. The culture was then washed with 25 times the volume of cells using M9 and centrifuged at 3,488 g. The pellet was re-suspended in the initial volume of M9 with 50 µg/mL kanamycin and an aliquot of cells was spread on custom-made agarose pads [1% agarose in M9 solidified for 10 min on a glass slide (VWR) as a slab with 1 mm thickness] and imaged immediately. The culture was kept in parallel at 37 °C at 200 rpm for normal growth. After 45 min, an aliquot of the culture was imaged in the same way. For experiments using cephalexin, 100 µg/ml cephalexin (Sigma-Aldrich) was added to the culture 1.5 h post-induction. Cells were grown for an additional 1.5 h and then labeled with SpyTag002-mClover3 as above.

For steady-state binding experiments (Fig. 4b), the cells were grown and induced as above. For labeling, cells were incubated in M9 with 1 µM SpyTag002-sfGFP or SpyTag002 DA-sfGFP on ice for 45 min, washed as described above and then imaged.

Microscopy
Cells were imaged on a CoolLED pE-1000 inverted, epifluorescence Nikon Eclipse Ti microscope using a 100× NA1.4 PlanApo oil immersion objective fitted with LED excitation source (Lumencor SpectraX), a Hamamatsu Orca-Flash 4.0 camera and encased in a chamber (Okolabs) set to 37 °C. Cells stained with sfGFP or mClover3 were imaged using λ_{exc} 485 nm and λ_{em} 510-555 nm. Images were analyzed using Nikon NIS Elements viewer version 4.20 and ImageJ 1.46r (National Institutes of Health) software. For Figure 4b, cells were stained, imaged and analyzed under identical settings. For Figures 4c and 4d, contrast was adjusted for each image to optimize visibility of the fluorescent protein distribution.

We used MicrobeTracker[S18] to determine the contours of each cell using brightfield images and the fluorescent intensities. From the long axis, one end was defined as the origin (Fractional length of cell = 0) arbitrarily, based on the orientation in the frame. The sum of the fluorescent intensities of a transverse 1 pixel-wide slice perpendicular to the *E. coli* long axis was divided by the number of pixels in that slice. The data were interpolated in 20 bins to account for the different cell length (mean cell length was 20.3 pixels). The value for each bin was divided by the median value of all the bins for that cell, to give the Relative Intensity, so that the cellular distribution of the fluorescence could be compared between cells with differing total staining intensity. The mean Relative Intensity for all cells in the field of view is plotted, based on at least 25 cells analyzed per condition. 95% confidence intervals on this mean Relative Intensity were calculated parametrically using MATLAB 2016b (MathWorks) with the tinv function and plotted using MATLAB.

For imaging of movies, *E. coli* BL21-T7Express cells were transformed with intimin-SpyCatcher002 and grown in M9 medium overnight at 37 °C with 50 µg/mL kanamycin. Cells were diluted in M9 to give an OD_{600} equal to 0.05. The cells were then grown to OD_{600} 0.3 and induced with 0.42 mM IPTG for 3 h at 37 °C. SpyTag002-mClover3 was added to the cells to a final concentration of 2 µM for 20 min at 37 °C with shaking at 200 rpm. The cells were washed with 50-fold excess volume of M9, centrifuged at 3,488 g, and resuspended in 1 mL of M9. The cells were immediately spread on an M9-agarose pad sealed with an air-tight plastic gasket (Gene Frame, Thermo Fisher) to minimize drying and cells were thereafter imaged at 37 °C. For cells treated with cephalexin, 100 µg/mL cephalexin was added at the time of induction and maintained in all subsequent steps. Imaging was carried out using the microscope set-up described above, with fluorescent and brightfield images taken at 5 min intervals for 45 min. Time-lapse image analysis was carried out using ImageJ 1.46r and MATLAB. Images were corrected for drift in the x-y plane using MATLAB with MicrobeTracker. For the videos, background was subtracted from individual frames and brightness was adjusted to correct for photobleaching at later time-points. All changes were applied uniformly across the full field of view.

Western blotting

E. coli BL21-T7Express cells (NEB) were transformed with either intimin (no C-terminal myc-tag), intimin-SpyCatcher002 (containing a C-terminal myc-tag), or intimin-SpyCatcher002 EQ (containing a C-terminal myc-tag) and grown overnight in LB with 50 µg/mL kanamycin at 37 °C at 200 rpm. In the morning, 250 µL culture was diluted into 10 mL M9 media with 50 µg/mL kanamycin and grown to OD_{600} 0.2-0.4, before inducing with 0.42 mM IPTG at 30 °C at 200 rpm. After 2 h, the culture was centrifuged at 3,488 g for 5 min, washed with 25 times the volume of cells using PBS pH 7.4 and centrifuged at 3,488 g for 5 min. The cells were resuspended in 100 µL PBS pH 7.4 with either 10 µL PBS pH 7.4 or 10 µL 77 µM biotinylated Avitag-SpyTag002-MBP in PBS pH 7.4. Cells were incubated at 37 °C with shaking at 1,000 rpm using an Eppendorf ThermoMixer for 20 min. Cells were washed three times using PBS pH 7.4 and centrifugation at 3,488 g for 5 min. Cells were resuspended in 100 µL lysis buffer [TBS and 1% (v/v) Triton X-100 containing Complete mini EDTA-free protease inhibitor cocktail (Roche) plus 1 mM PMSF] on ice for 20 min, followed by centrifugation at 17,000 g for 10 min at 4 °C to pellet nuclei. The supernatant was removed and stored in 20 µL aliquots at -80 °C. Aliquots to be analyzed by blotting with streptavidin were thawed and 4 µL 6× SDS-PAGE loading buffer added, followed by heating at 95 °C for 6 min in a Bio-Rad C1000 thermal cycler. All subsequent steps were performed at 25 °C. SDS-PAGE was run on a 16% polyacrylamide gel and transferred to nitrocellulose membrane using program 2 on the iBlot Dry Blotting System (Life Technologies) according to the manufacturer's instructions. The membrane was incubated for 1 h in blocking buffer [TBS + 0.1% (v/v) Tween-20 + 3% (w/v) BSA], followed by 1 h with 1:5,000 dilution of streptavidin-horseradish peroxidase (Sigma-Aldrich) in blocking buffer. After washing the membrane in Wash buffer [TBS + 0.1% (v/v) Tween-20] four times (5 min incubations for each wash), horseradish peroxidase was detected using SuperSignal West Pico Chemiluminescent Substrate (Thermo Fisher) according to the manufacturer's instructions. Bands were measured using a Gel Doc XR imager in chemiluminescent mode, with Image Lab 5.0 software.

To blot for the myc tag, the blocked membrane was incubated for 1 h with a 1:1,000 dilution of mouse anti-myc tag (clone 9E10, Santa Cruz) antibody in blocking buffer. After washing the membrane in Wash buffer four times (5 min per wash), the membrane was incubated for 1 h with a 1:5,000 dilution of goat anti-mouse-HRP secondary antibody

(Sigma-Aldrich) in blocking buffer. After washing the membrane in Wash buffer 4 times (5 min incubations for each wash), HRP was detected as above.

Flow cytometry
E. coli BL21(DE3) (Stratagene) displaying intimin-SpyCatcher or intimin-SpyCatcher002 were grown overnight as above and induced in M9 medium at 30 ˚C for 2 h. The cells were washed in PBS and then 1 mL culture (OD_{600} ~0.7) was spun down at 5,000 g for 5 min. Cells were resuspended in 50 µL PBS in a 1.5 mL microcentrifuge tube with a dilution from a 100 µM stock of either SpyCatcher-mClover3 or SpyCatcher002-mClover3 in PBS. Cells were incubated at 37 ˚C for 5, 10 or 20 min, shaking at 600 rpm using an Eppendorf ThermoMixer. For the unlabeled control, no protein was added with a 20 min incubation. After the incubation, 1 mL PBS at 4 °C was added and cells were thereafter kept at 4 °C. Cells were spun at 10,000 g for 1 min, followed by washing three times with 1 ml PBS. The cells in 1 mL PBS were then analyzed by flow cytometry using a Bio-Rad S3e FACS machine. Settings were Forward Scatter (FSC) 400 V with 0.5 threshold, Side Scatter (SSC) 350 V, Fluorescence channel 1 (FL1, 488 nm excitation, 525 nm emission) 640 V. A gate was set to a FSC/SSC area corresponding to single *E. coli* cells and 100,000 events in this gate were collected. The resulting fsc3.1 files were analyzed in MATLAB. In order to read the fsc 3.1 files, the *fca_readfcs* function from MathWorks Exchange was used (https://uk.mathworks.com/matlabcentral/fileexchange/9608-fcs-data-reader). For the plot of intensities, the median of FL1 intensity was used after subtracting the median FL1 (resulting from autofluorescence) for the unlabeled control sample. For the logarithmic histogram plot, the data were binned into 301 intervals, each 4.7% larger than the previous interval.

The underlying research data can be accessed via e-mail request to the corresponding author.

Supporting references

[S1] B. Zakeri, J. O. Fierer, E. Celik, E. C. Chittock, U. Schwarz-Linek, V. T. Moy, M. Howarth, *Proc Natl Acad Sci U S A* **2012**, *109*, E690-697.
[S2] D. Beckett, E. Kovaleva, P. J. Schatz, *Protein Sci.* **1999**, *8*, 921-929.
[S3] J. Chiu, P. E. March, R. Lee, D. Tillett, *Nucleic Acids Res* **2004**, *32*, e174.
[S4] G. Veggiani, T. Nakamura, M. D. Brenner, R. V. Gayet, J. Yan, C. V. Robinson, M. Howarth, *Proc Natl Acad Sci U S A* **2016**, *113*, 1202-1207.
[S5] M. Friedman, A. Orlova, E. Johansson, T. L. Eriksson, I. Hoiden-Guthenberg, V. Tolmachev, F. Y. Nilsson, S. Stahl, *J Mol Biol* **2008**, *376*, 1388-1402.
[S6] J. E. Tropea, S. Cherry, D. S. Waugh, *Methods Mol Biol* **2009**, *498*, 297-307.
[S7] J. D. Pedelacq, S. Cabantous, T. Tran, T. C. Terwilliger, G. S. Waldo, *Nat Biotech* **2006**, *24*, 79-88.
[S8] Y. Xia, W. Chu, Q. Qi, L. Xun, *Nucleic Acids Res* **2015**, *43*, e12.
[S9] B. T. Bajar, A. J. Lam, R. K. Badiee, Y. H. Oh, J. Chu, X. X. Zhou, N. Kim, B. B. Kim, M. Chung, A. L. Yablonovitch, B. F. Cruz, K. Kulalert, J. J. Tao, T. Meyer, X. D. Su, M. Z. Lin, *Nat Methods* **2016**, *13*, 993-996.
[S10] V. Salema, E. Marin, R. Martinez-Arteaga, D. Ruano-Gallego, S. Fraile, Y. Margolles, X. Teira, C. Gutierrez, G. Bodelon, L. A. Fernandez, *PLoS ONE* **2013**, *8*, e75126.
[S11] J. Engberg, P. S. Andersen, L. K. Nielsen, M. Dziegiel, L. K. Johansen, B. Albrechtsen, *Mol Biotech* **1996**, *6*, 287-310.
[S12] D. Steiner, P. Forrer, M. T. Stumpp, A. Pluckthun, *Nat Biotech* **2006**, *24*, 823-831.
[S13] S. Jung, A. Honegger, A. Pluckthun, *J Mol Biol* **1999**, *294*, 163-180.
[S14] L. Dumon-Seignovert, G. Cariot, L. Vuillard, *Protein Expr Purif* **2004**, *37*, 203-206.

[S15] M. Fairhead, M. Howarth, *Methods Mol Biol* **2015**, *1266*, 171-184.
[S16] M. Oke, L. G. Carter, K. A. Johnson, H. Liu, S. A. McMahon, X. Yan, M. Kerou, N. D. Weikart, N. Kadi, M. A. Sheikh, S. Schmelz, M. Dorward, M. Zawadzki, C. Cozens, H. Falconer, H. Powers, I. M. Overton, C. A. van Niekerk, X. Peng, P. Patel, R. A. Garrett, D. Prangishvili, C. H. Botting, P. J. Coote, D. T. Dryden, G. J. Barton, U. Schwarz-Linek, G. L. Challis, G. L. Taylor, M. F. White, J. H. Naismith, *J Struct Funct Genomics* **2010**, *11*, 167-180.
[S17] L. Li, J. O. Fierer, T. A. Rapoport, M. Howarth, *J Mol Biol* **2014**, *426*, 309-317.
[S18] E. C. Garner, *Mol Microbiol* **2011**, *80*, 577-579.

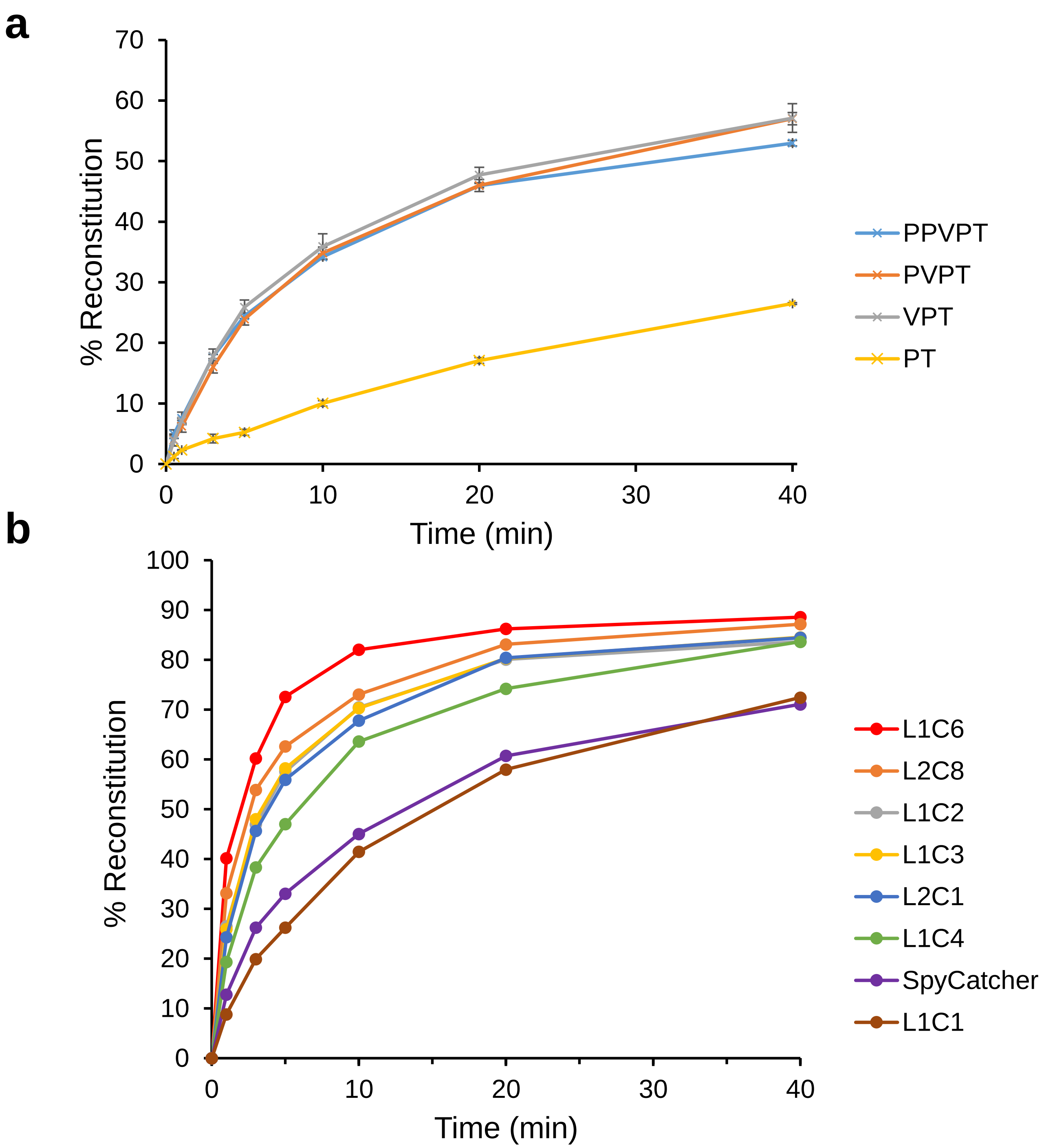

Figure S1: Testing reaction speed of selected clones. a) Reaction analysis of deletions of the SpyTag N-terminal library's most reactive variant (PPVPT-SpyTag-MBP). Time-course of SpyCatcher reacting with deletion variants, with each protein at 1 μM at 25 °C in PBS pH 7.5, after analysis by SDS-PAGE with Coomassie staining. The indicated N-terminal sequence is followed by -IVMVDAYKPTK. The data show the mean of reactions carried out in triplicate ± 1 s.d.; some error bars are too small to be visible. b) Reaction time-courses of phage-selected SpyCatcher variants. SpyTag-MBP was incubated with SpyCatcher and selected variants, with each protein at 1 μM at 25 °C in PBS pH 7.5. Reaction was analyzed after boiling by SDS-PAGE with Coomassie staining. The data show the means of duplicate reactions.

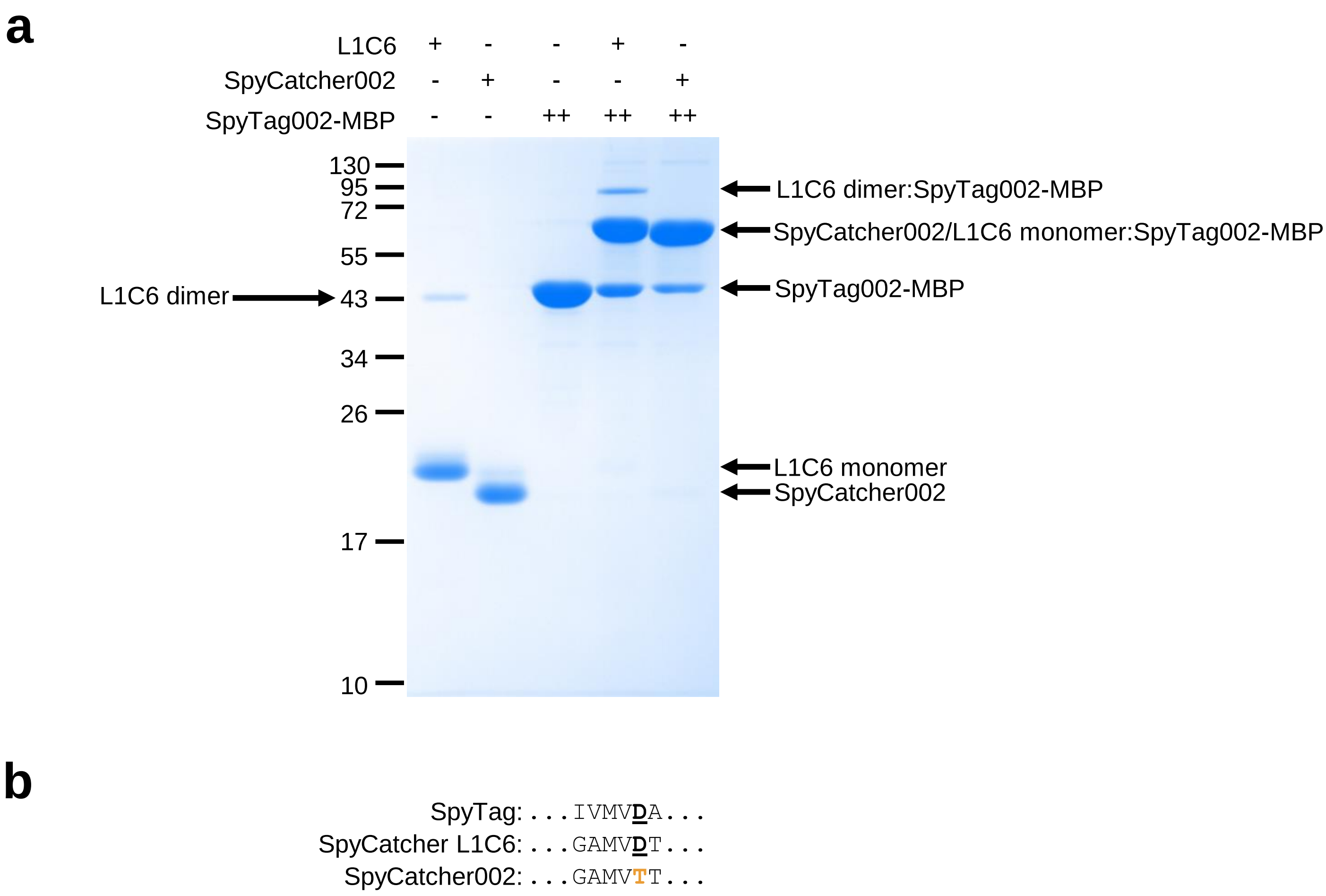

Figure S2: Optimization of phage-selected SpyCatcher variant. a) Self-reaction of L1C6 SpyCatcher variant was blocked in SpyCatcher002. L1C6 and SpyCatcher002 were analyzed in isolation or after reaction with SpyTag002-MBP by SDS-PAGE with Coomassie staining. A small fraction of covalent L1C6 dimer is marked, as well as the product from L1C6 dimer reacting with SpyTag002-MBP. Reaction conditions: 10 µM (+) SpyCatcher variant, 13 µM (++) SpyTag002-MBP, PBS pH 7.5 at 25 ˚C for 1 h. b) Alignment of part of the amino acid sequence of SpyTag with the N-terminus of SpyCatcher L1C6. L1C6 D2T (SpyCatcher002) was created, preventing self-reaction.

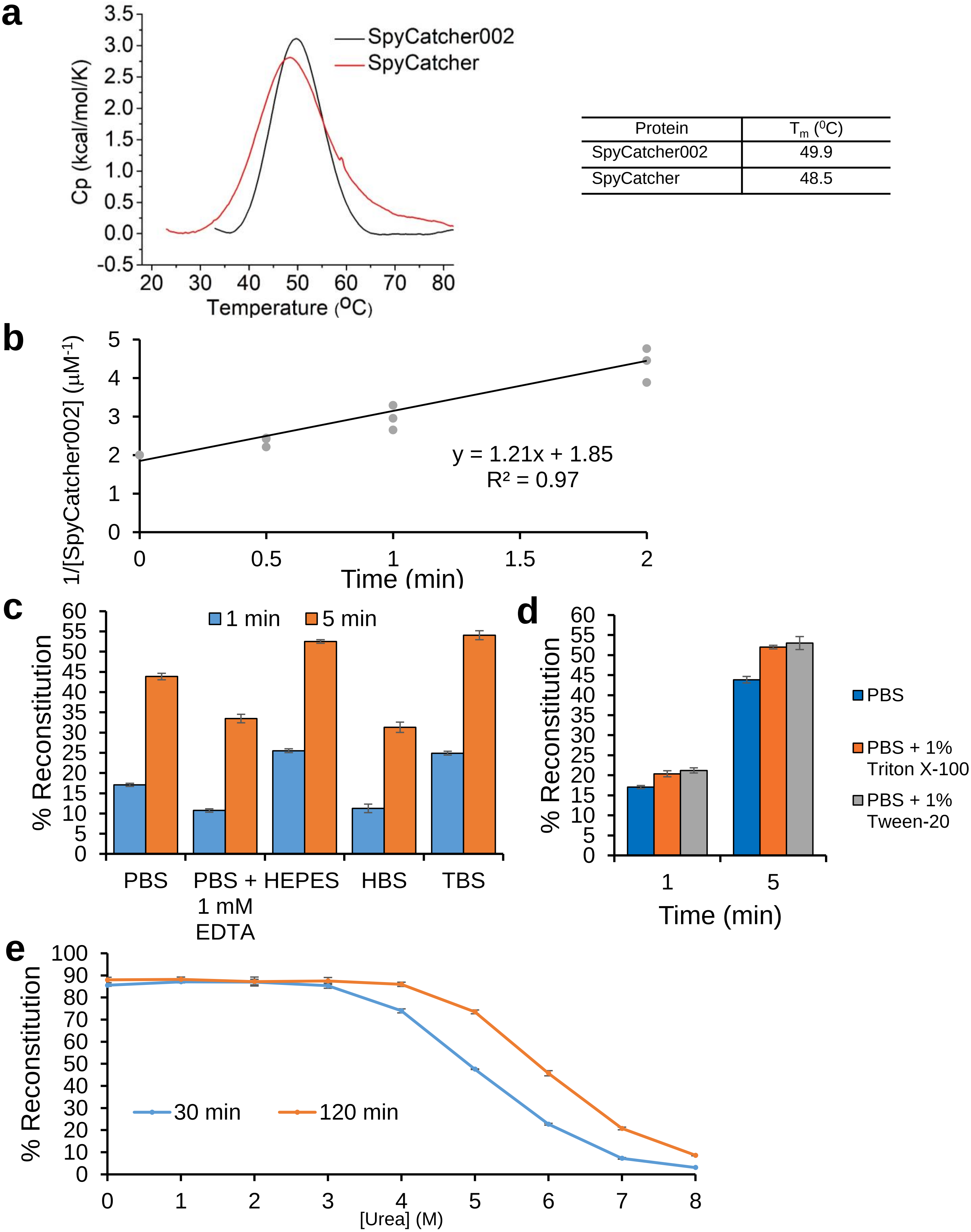

Protein	T_m (°C)
SpyCatcher002	49.9
SpyCatcher	48.5

Figure S3: Characterization of selected pair. a) DSC of SpyCatcher (red) and SpyCatcher002 (black). T_m values are inset. Full Width Half Maximum: SpyCatcher 16 °C, SpyCatcher002 12 °C. b) Quantifying rate constant for SpyCatcher002 reacting with SpyTag002-MBP; n = 3, each point shown. 0.5 μM of each protein was in succinate-phosphate-glycine buffer at pH 7.0, 25 ˚C. The equation for the trend-line and correlation coefficient are shown. c) Buffer-dependence of SpyCatcher002 and SpyTag002-MBP reaction at 25 °C and pH 7.5 with PBS, PBS + 1 mM EDTA, 50 mM HEPES, 50 mM HEPES-buffered saline (HBS), or Tris-buffered saline (TBS) for 1 or 5 min at 25 °C. d) Detergent-dependence of reaction as in c) with no detergent (PBS), PBS with 1% Triton X-100, or PBS with 1% Tween-20. e) Urea dependence of SpyCatcher002 and SpyTag002-MBP reaction at 25 °C and pH 7.5 in PBS for 30 or 120 min at 25 °C. c/d/e show mean of triplicate ± 1 s.d.; some error bars are too small to be visible.

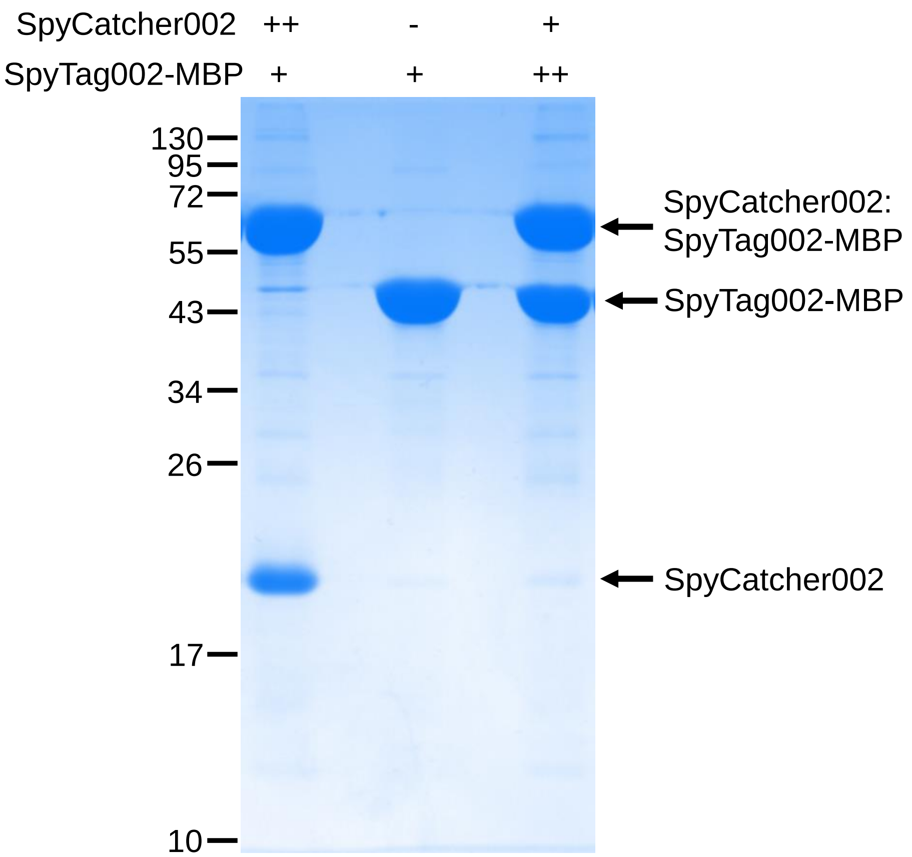

Figure S4: Testing the reaction of SpyCatcher002/SpyTag002 to completion. SpyCatcher002 was incubated with SpyTag002-MBP in succinate-phosphate-glycine buffer pH 7.0 for 1 h at 25 °C before analysis by SDS-PAGE and Coomassie staining. Proteins were at 10 μM (+) or 20 μM (++).

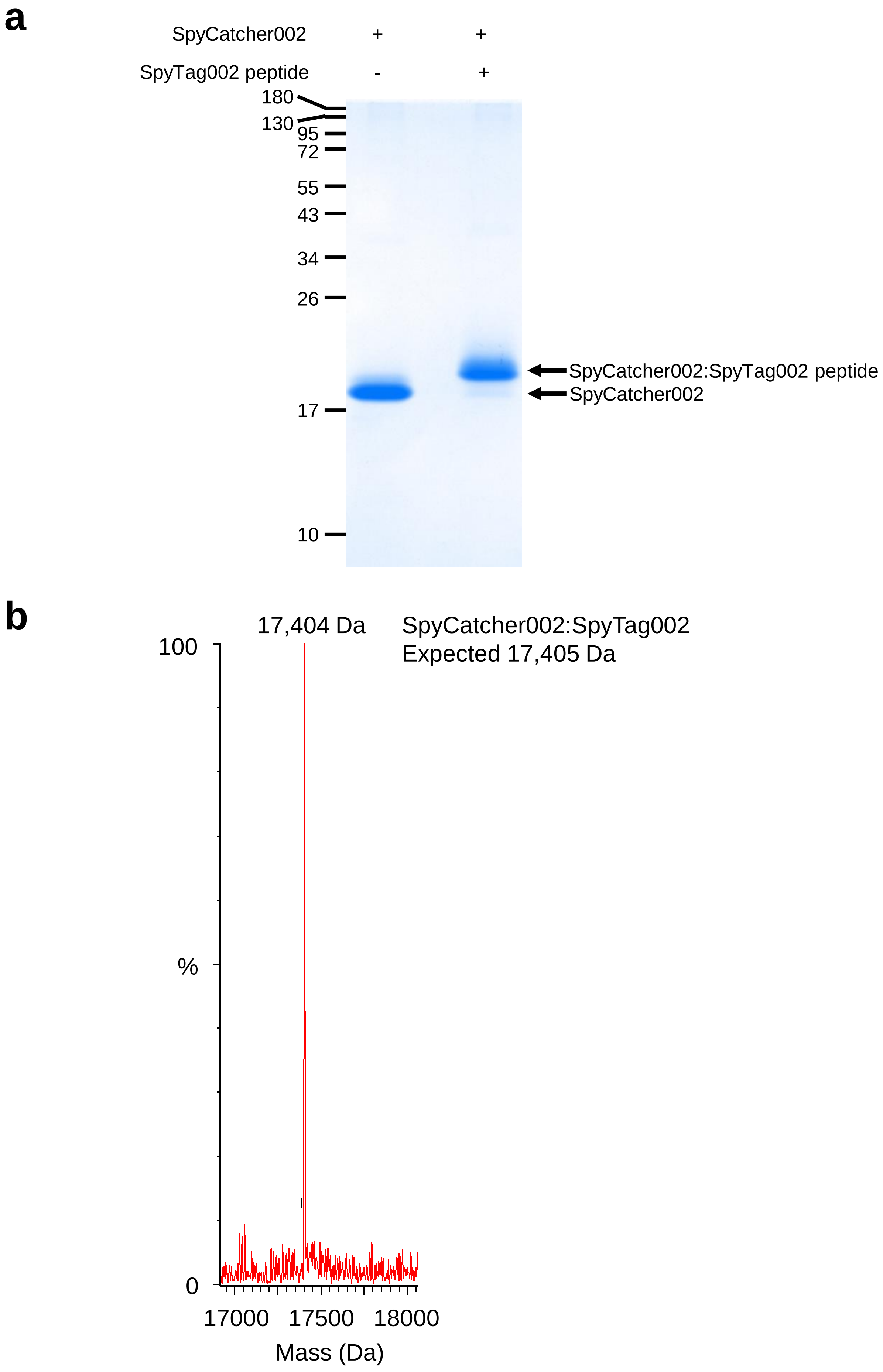

Figure S5: Characterization of isopeptide bond formation by mass spectrometry. a) Reaction of SpyCatcher002 with the SpyTag002 peptide was analyzed by 18% SDS-PAGE with Coomassie staining. Unreacted SpyTag002 peptide (1.8 kDa) was not resolved from the dye front. b) Electrospray Ionization Mass Spectrometry of the SpyCatcher002:SpyTag002 peptide product with the expected loss of H_2O upon reaction.

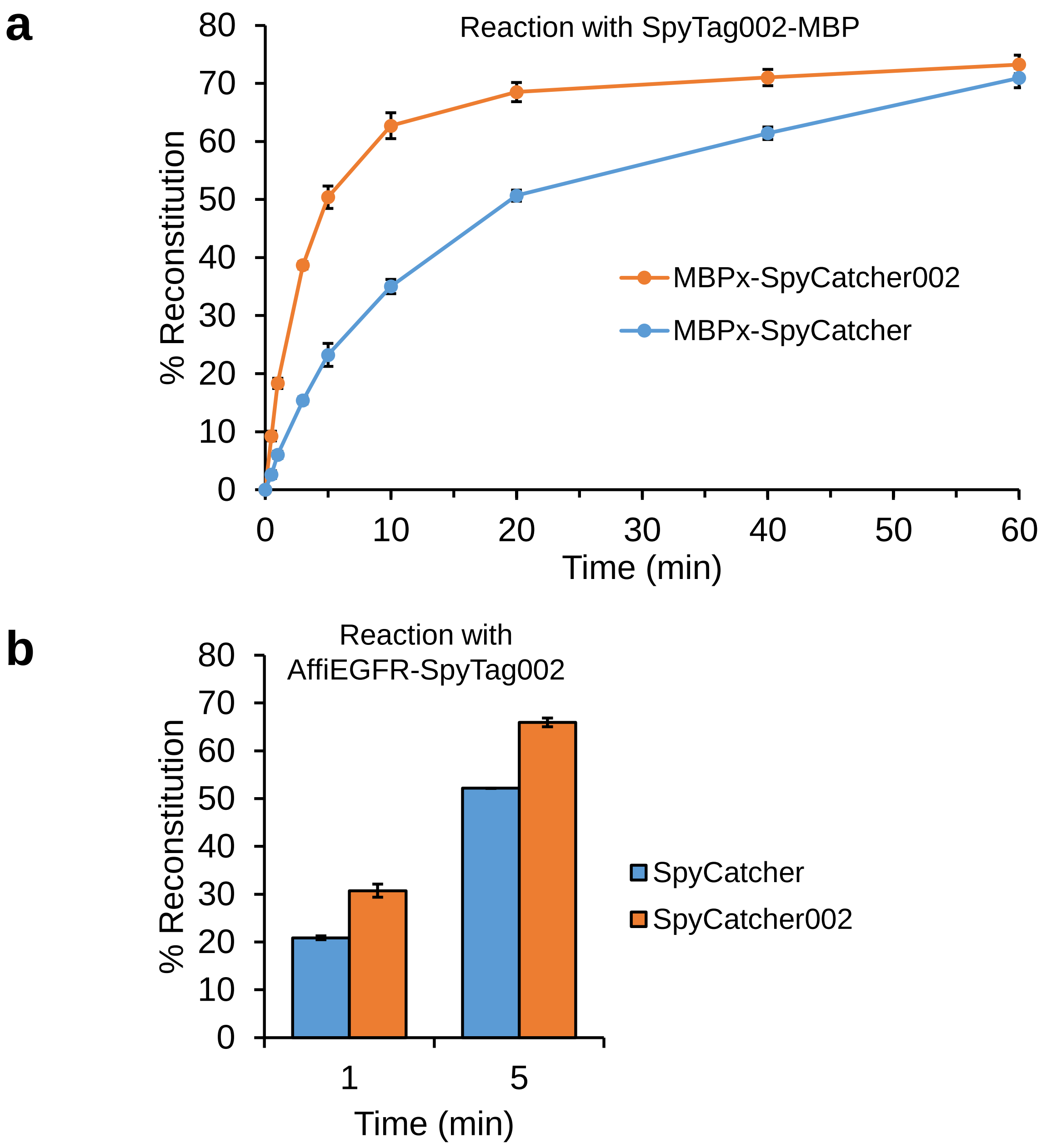

Figure S6: Tolerance of SpyCatcher002 and SpyTag002 to fusion at different termini. a) Improved reactivity of SpyCatcher002 over SpyCatcher was retained when a protein was fused to the N-terminus. Time-course of MBPx-SpyCatcher and MBPx-SpyCatcher002 reacting with SpyTag002-MBP, with each protein at 0.5 μM at 25 °C in PBS pH 7.5, analyzed after boiling by SDS-PAGE with Coomassie staining. b) Improved reactivity of SpyCatcher002 over SpyCatcher was retained when SpyTag002 was at the C-terminus. AffiEGFR-SpyTag002 was incubated with SpyCatcher or SpyCatcher002 for 1 or 5 min, with each protein at 2 μM at 25 °C in PBS pH 7.5 and analyzed by SDS-PAGE with Coomassie staining. Data show the mean of reactions carried out in triplicate ± 1 s.d.; some error bars are too small to be visible.

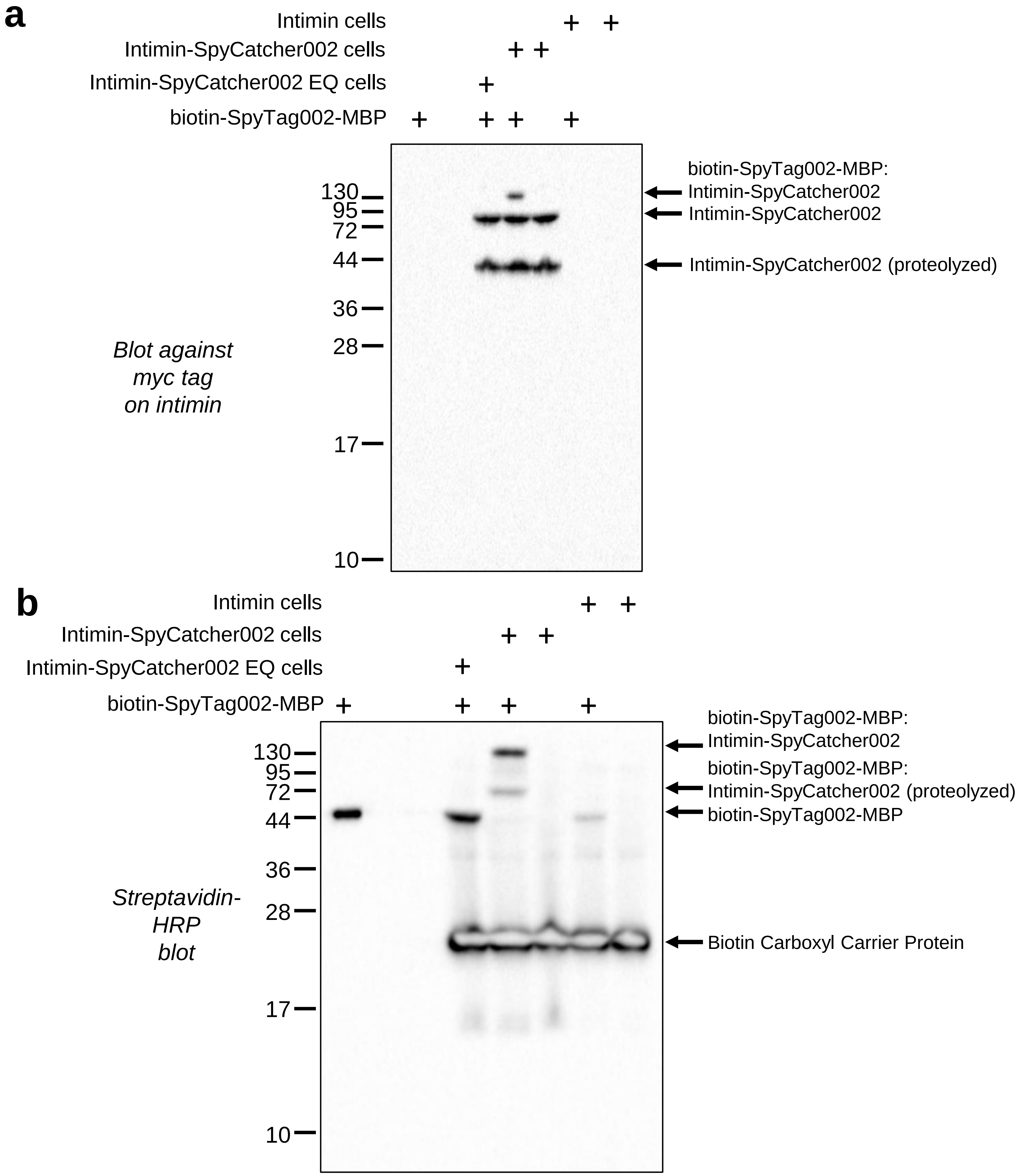

Figure S7: SpyCatcher002 and SpyTag002 reacted specifically on cells. *E. coli* expressing intimin (without myc tag), intimin-SpyCatcher002 or intimin-SpyCatcher002 EQ (both bearing a myc tag) were analyzed by Western blotting ± biotin-SpyTag002-MBP incubation. Purified biotin-SpyTag002-MBP was run as a control. a) Lysates were probed with anti-myc tag antibody. No new bands were seen when comparing SpyCatcher002 and SpyCatcher002 EQ, unless the SpyTag002 target was present. Intimin degradation, most likely in the β-barrel domain, was seen for both SpyCatcher002 and SpyCatcher002 EQ. b) Lysates were probed with streptavidin-HRP. Covalent product was only formed when biotin-SpyTag002-MBP reacted with cells expressing intimin-SpyCatcher002. Some of the biotin-SpyTag002-MBP was not fully removed from cells by the washing. Biotin Carboxyl Carrier Protein is an endogenous biotinylated protein of *E. coli* and serves as a loading control.

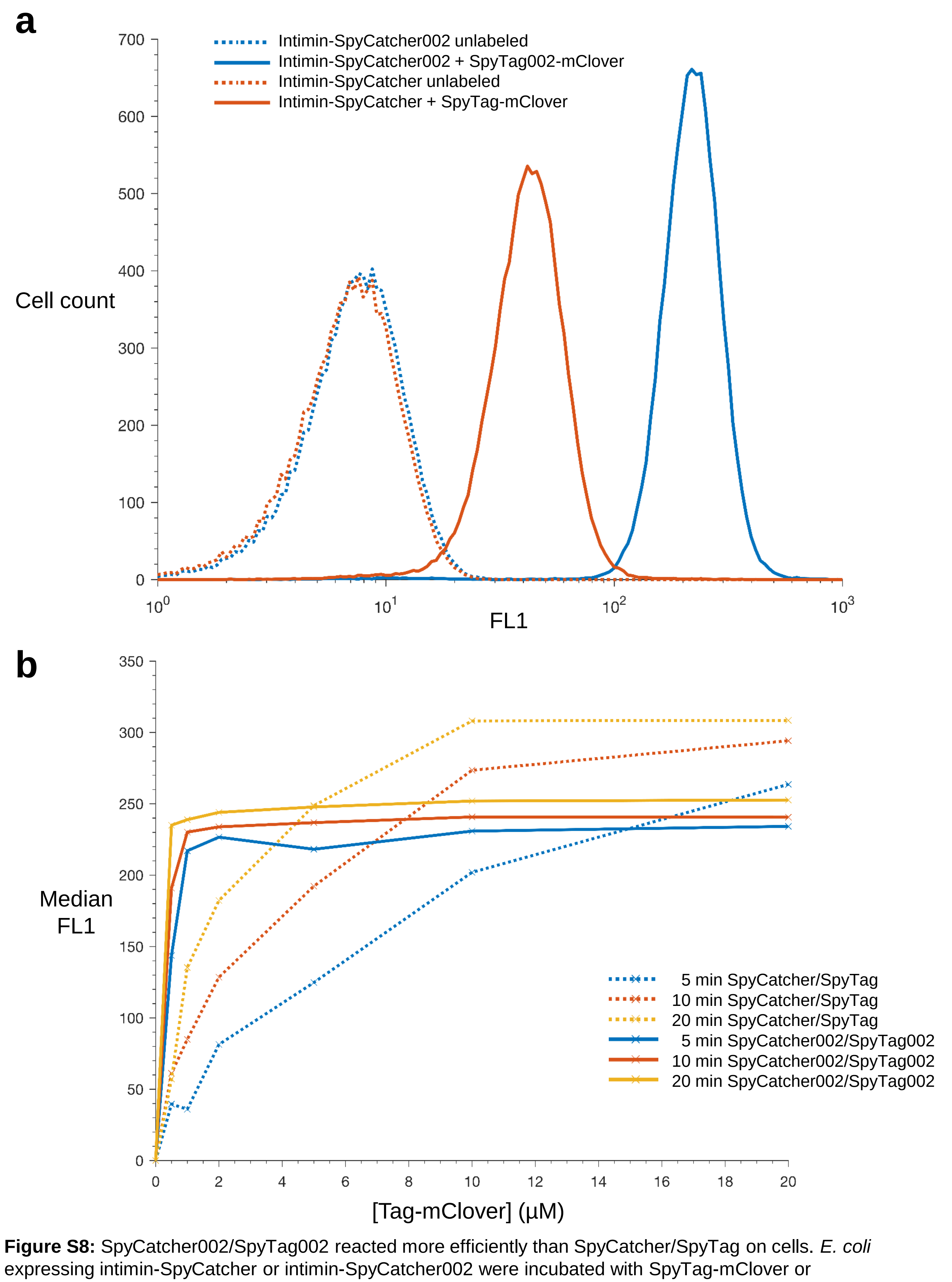

Figure S8: SpyCatcher002/SpyTag002 reacted more efficiently than SpyCatcher/SpyTag on cells. *E. coli* expressing intimin-SpyCatcher or intimin-SpyCatcher002 were incubated with SpyTag-mClover or SpyTag002-mClover. Cell staining was analyzed by flow cytometry. a) Cell count versus fluorescence intensity for cells unlabeled or incubated with 1 µM SpyTag-mClover or SpyTag002-mClover for 5 min. b) Plot of median cell fluorescence after 5, 10 or 20 min with the indicated concentration of SpyTag-mClover or SpyTag002-mClover, with the autofluorescence signal subtracted.

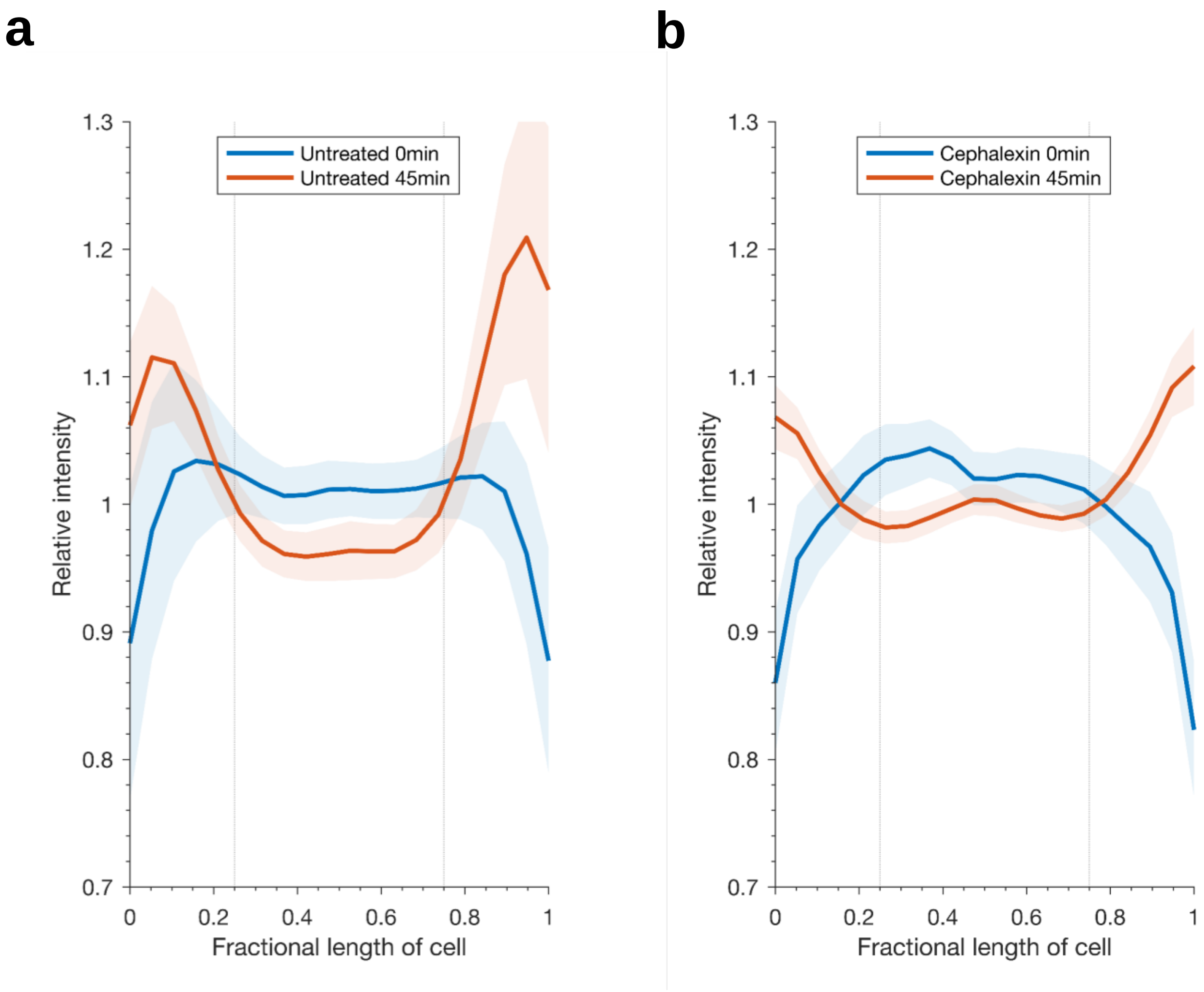

Figure S9: Quantification of polar localization of intimin-SpyCatcher002. *E. coli* expressing intimin-SpyCatcher002 were labeled with SpyTag002-mClover and then imaged immediately (0 min) or incubated for 45 min at 37 °C, as in Fig. 4. Cells were untreated in a) or treated with cephalexin in b). Fluorescence was quantified along the length of the cell, with the median intensity for each cell normalized to a value of 1. The curve represents the median value for all the cells imaged under a given condition, with the shaded area representing 95% confidence intervals. Dotted lines are drawn 25% and 75% along the cell.

Movie S1: Fluorescent microscopy in green fluorescent protein channel (grayscale) over 45 min of *E. coli* expressing intimin-SpyCatcher002 and labeled with SpyTag002-mClover. Scale-bar 2 µm.

Movie S2: Bright-field microscopy over 45 min of *E. coli* expressing intimin-SpyCatcher002 and labeled with SpyTag002-mClover. Scale-bar 2 µm.

Movie S3: Fluorescent microscopy in green fluorescent protein channel (grayscale) over 45 min of cephalexin-treated *E. coli* expressing intimin-SpyCatcher002 and labeled with SpyTag002-mClover. Scale-bar 2 µm.

Movie S4: Bright-field microscopy over 45 min of cephalexin-treated *E. coli* expressing intimin-SpyCatcher002 and labeled with SpyTag002-mClover. Scale-bar 2 µm.
